# Is This Contrast? Is This Blood? An Agreement Study on Post-thrombectomy Computed Tomography Scans

**DOI:** 10.3389/fneur.2020.593098

**Published:** 2020-12-22

**Authors:** Ronda Lun, Gregory B. Walker, Adrien Guenego, Mohammed Kassab, Eduardo Portela, Vignan Yogendrakumar, George Medvedev, Ken Wong, Michel Shamy, Dar Dowlatshahi, Robert Fahed

**Affiliations:** ^1^Ottawa Stroke Program, Department of Medicine (Neurology), University of Ottawa, Ottawa, ON, Canada; ^2^Division of Neurology, Fraser Health Authority, Royal Columbian Hospital, New Westminster, BC, Canada; ^3^Department of Interventional Neuroradiology, Erasme University Hospital, Brussels, Belgium; ^4^Division of Neurosurgery, Department of Surgery, University of Ottawa, Ottawa, ON, Canada; ^5^Interventional Neuroradiology, Department of Medical Imaging, University of Ottawa, Ottawa, ON, Canada; ^6^Division of Medical Imaging, Fraser Health Authority, Royal Columbian Hospital, New Westminster, BC, Canada; ^7^Ottawa Hospital Research Institute, Ottawa, ON, Canada

**Keywords:** stroke, ischemic stroke, thrombectomy, hemorrhage, cerebral, agreement, reliability

## Abstract

**Background:** Hemorrhagic transformation after acute ischemic stroke is a dreaded and severe complication of thrombolysis and thrombectomy. However, its detection on post-thrombectomy conventional non-contrast computed tomography (CT) scan can be complicated by the frequent (and sometimes concomitant) presence of contrast, resulting in changes in management.

**Aims:** Our objective was to assess the inter- and intra-rater reliability for the detection of blood and/or contrast on day-1 post-thrombectomy CT scans.

**Methods:** A total of 18 raters across 3 different specialties independently examined 30 post-thrombectomy CT scans selected from the Aspiration vs. STEnt-Retriever (ASTER) trial. They were asked to judge the presence of blood and contrast. Thirty days later, the same 18 raters again independently judged the 30 scans, in randomized order. Agreement was measured with Fleiss' and Cohen's *K* statistics.

**Results:** Overall agreement on blood and/ or contrast presence was only fair, *k* = 0.291 (95% CI = 0.273–0.309). There were 0 scans with consensus among the 18 readers on the presence of blood and/or contrast. However, intra-rater global agreement across all 18 physicians was relatively high, with a median kappa value of 0.675. This intra-rater consistency was seen across all specialties, regardless of level of training.

**Conclusion:** Physician judgment for the presence of blood and/or contrast on day-1 post-thrombectomy non-contrast CT scan shows limited inter-observer reliability. Advanced imaging modalities may then be warranted for challenging clinical cases.

## Introduction

Endovascular thrombectomy (EVT) has become the standard of care for patients with acute ischemic stroke (AIS) secondary to large vessel occlusions (1). One of the major complications after AIS is hemorrhagic transformation (HT), reported to be up to 35% after EVT (2). However, arterial injection of iodine contrast during EVT may mimic the appearance of HT, due to its hyper-dense appearance on follow-up conventional non-contrast CT (NCCT) (3). Inaccurate identification of HT could delay necessary treatments such as antiplatelet or anticoagulant therapy, and potentially result in misdiagnosis of HT in future EVT trials. In this study, we aimed to evaluate inter-rater and intra-rater reliability for detection of HT and contrast staining (CS) on NCCT in EVT patients.

## Methods

We analyzed imaging data from the “Aspiration vs. STEnt-Retriever” (ASTER) trial (4). Access to the data can be obtained through formal proposal to the authors of the study.

A total of 30 NCCT scans performed 24–36 h after EVT were selected from the ASTER database with a roughly equal distribution of scans with HT, scans with CS, scans with both HT and CS, and scans with no HT or CS (i.e., approximately 6–8 scans in each subcategory). The studies were identified as such in the core laboratory of the ASTER study, which is composed of four attending physicians with 5–20 years of experience in neuroradiology. All selected cases of HT were parenchymal; we did not assess for the detection of subarachnoid or extra-cranial hemorrhage. Additional clinical details of the study are outlined elsewhere (5).

Eighteen raters from 3 tertiary stroke centers were recruited for independent interpretation of studies: 6 stroke neurologists, 6 interventional neuroradiologists (INR), and 6 diagnostic neuroradiologists (DNR), as they are all involved in multidisciplinary decisions for stroke patients. Within each specialty, there were 3 junior level physicians and 3 senior level physicians. Junior physicians were defined as fellows with <2 years of experience, and senior physicians were defined as staff physicians with more than 5 years of independent practice experience. To evaluate inter-rater reliability, the raters were asked to independently evaluate each NCCT for (1) presence of hemorrhage and (2) presence of contrast. To evaluate intra-rater reliability, the same independent raters were then asked to repeat the study assessments 1 month later, with the study order randomized. The raters had access to basic clinical information, including basic demographics, treatment status with thrombolysis, final thrombolysis in cerebral infarction recanalization score, time from symptom onset to recanalization, and day-1 National Institutes of Health Stroke Scale score (5).

The raters' dichotomized (yes/no) answers were transformed into a “global judgment” score of whether there was blood, contrast, or a combination of both. Fleiss' kappa was run to determine if there was inter-rater agreement. Intra-rater reliability was also assessed with Cohen's unweighted kappa values. The median number of times a rater's answer changed between the two assessments was calculated. All statistics were performed using SPSS v26.0 (IBM, Armonk, NY). Graphs were generated using GraphPad Prism v8.3.1. Levels of agreement were defined according to Landis and Koch: slight (0.00–0.20), fair (0.20–0.40), moderate (0.40–0.60), substantial (0.60–0.80), and excellent (>0.80) (6).

## Results

**Figure 1:**
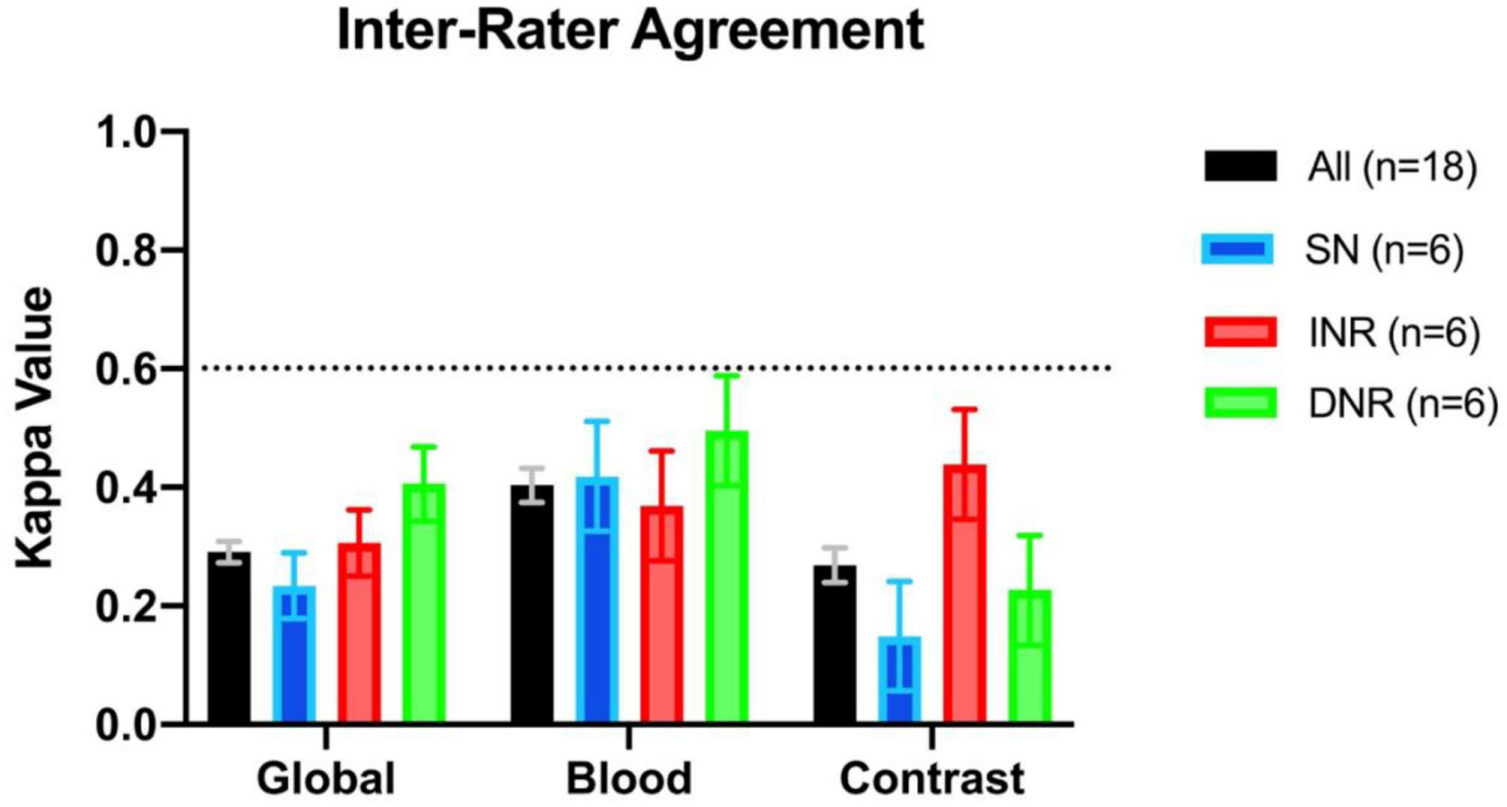
Interrater agreement displayed as kappa values across specialties for assessment of the presence vs. absence of blood, contrast, or presence of both (i.e., global assessment). SN, stroke neurologist; INR, interventional neuroradiologist; DNR, diagnostic neuroradiologist.

**Figure 2:**
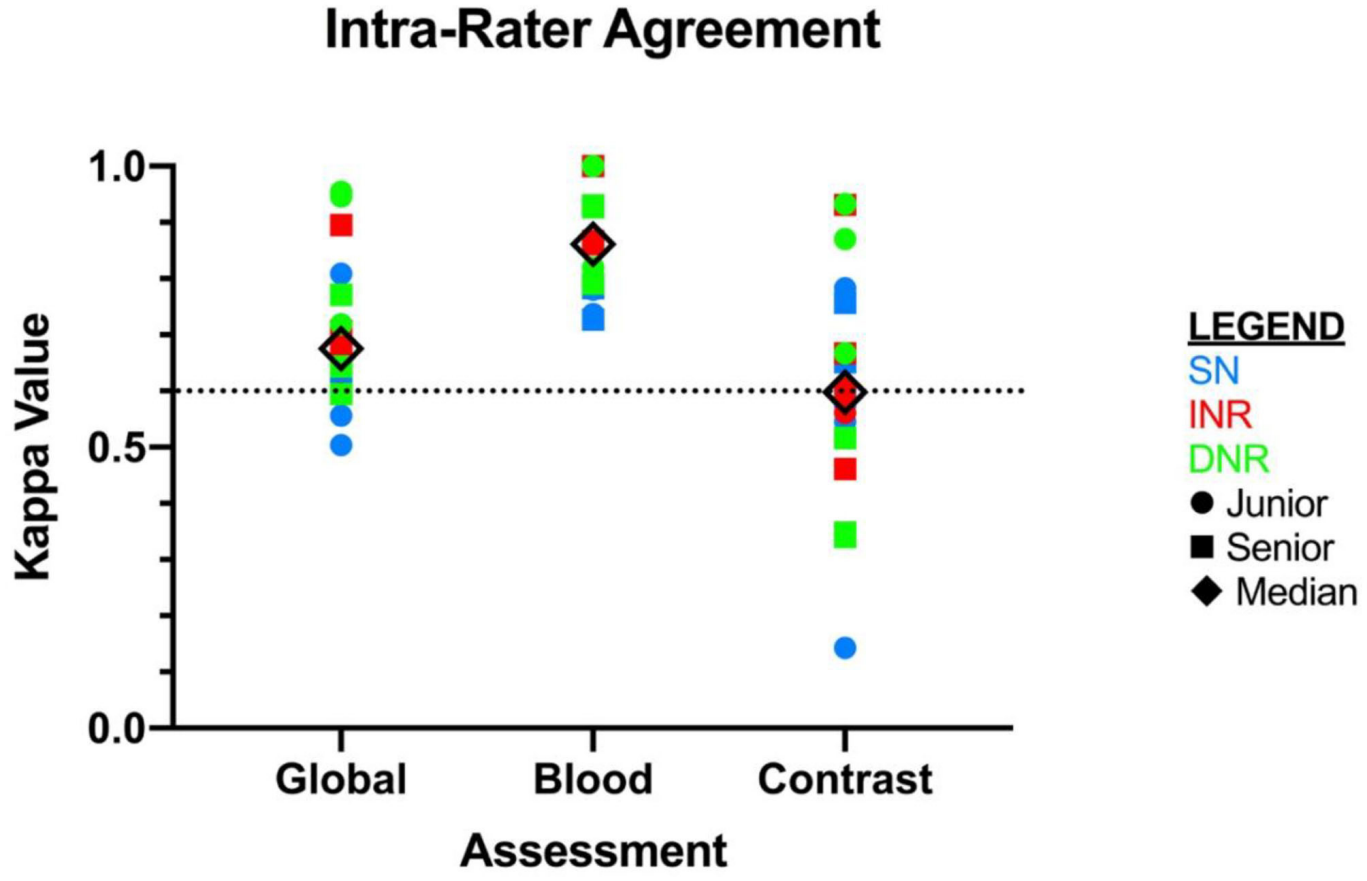
Intra-rater agreement displayed as kappa values for each independent rater. *X*-axis is displayed by type of assessment: global, blood, and contrast. SN, stroke neurologist; INR, interventional neuroradiologist; DNR, diagnostic neuroradiologist.

Inter-rater agreement on the detection of blood was moderate, *k* = 0.404 (95% CI = 0.375–0.432) (see Figure 1). There were 3/30 scans on which the 18 raters agreed upon the presence of blood, and 2/30 scans on which there was unanimous agreement on the absence of blood. Intra-rater agreement was at least substantial for the detection of blood across all specialties and levels of training, and 3 raters had perfect intra-rater agreement (median *k* value of 0.861) (Figure 2). The median number of changes of judgment between both readings was 2 (Table 1).

**Table 1:** Inter-rater agreement across specialties, displayed as Fleiss' Kappa values, with 95% confidence intervals.

	**Inter-Rater Agreement (1st Reading)**		
	**Global judgment**	**Blood (Yes/No)**	**Contrast (Yes/No)**
All (*n* = 18)	0.291 [0.273–0.309]	0.404 [0.375–0.432]	0.269 [0.240–0.298]
Stroke neurologists (*n* = 6)	0.234 [0.179–0.290]	0.418 [0.326–0.511]	0.149 [0.057–0.241]
Interventional neuroradiologists (*n* = 6)	0.306 [0.250–0.362]	0.369 [0.276–0.461]	0.439 [0.346–0.531]
Diagnostic neuroradiologists (*n* = 6)	0.406 [0.343–0.468]	0.495 [0.403–0.588]	0.227 [0.134–0.319]

The inter-rater agreement for the detection of CS was only fair, *k* = 0.269 (95% CI = 0.240–0.298) (Figure 1). There were 0 scans on which all raters agreed upon the presence of contrast, and only 2/30 scans on which they unanimously agreed on the absence of contrast. The INR physicians collectively had the highest level of agreement, which nevertheless remained moderate (*k* = 0.439, 95% CI = 0.346–0.531) (Table 1). Intra-rater agreement was below substantial for 9/18 raters, and no rater reached perfect intra-rater agreement (median *k* value of 0.598) (Figure 2). The median number of changes of judgment per physician was 4.5. Detailed kappa values for each rater are outlined in Table 2.

**Table 2:** Intra-rater agreements for 18 independent raters, displayed as Fleiss' Kappa values, with 95% confidence intervals.

	**Intra-Rater Agreement**		
	**Global judgment**	**Blood (Yes/No)**	**Contrast (Yes/No)**
Junior stroke neurologist #1	0.504 [0.271–0.767]	**0.737 [0.497–0.978]**	0.545 [0.251–0.840]
Junior stroke neurologist #2	**0.809 [0.604–1.000]**	**0.789 [0.562–1.000]**	**0.783 [0.364–1.000]**
Junior stroke neurologist #3	0.556 [0.293–0.820]	**0.780 [0.545–1.000]**	0.143 [−0.471–0.757]
Senior stroke neurologist #1	**0.610 [0.378–0.841]**	**0.783 [0.549–1.000]**	0.590 [0.262–0.918]
Senior stroke neurologist #2	**0.667 [0.450–0.883]**	**0.727 [0.478–0.976]**	**0.757 [0.496–1.000]**
Senior stroke neurologist #3	**0.668 [0.452–0.883]**	**0.867 [0.666–1.000]**	**0.651 [0.372–0.930]**
Junior interventional neuroradiologist #1	**0.681 [0.474–0.888]**	**0.862 [0.677–1.000]**	**0.605 [0.323–0.888]**
Junior interventional neuroradiologist #2	**0.707 [0.497–0.917]**	**0.861 [0.675–1.000]**	0.561 [0.210–0.912]
Junior interventional neuroradiologist #3	0.596 [0.375–0.817]	**0.795 [0.574–1.000]**	0.587 [0.292–0.883]
Senior interventional neuroradiologist #1	**0.895 [0.754–1.000]**	**0.861 [0.675–1.000]**	**0.931 [0.799–1.000]**
Senior interventional neuroradiologist #2	**0.707 [0.497–0.917]**	**0.867 [0.688–1.000]**	**0.667 [0.363–0.971]**
Senior Interventional Neuroradiologist #3	**0.657 [0.435–0.880]**	**1**	0.461 [0.112–0.811]
Junior diagnostic neuroradiologist #1	**0.955 [0.868–1.000]**	**1**	**0.933 [0.805–1.000]**
Junior diagnostic neuroradiologist #2	**0.946 [0.840–1.000]**	**1**	**0.870 [0.618–1.000]**
Junior diagnostic neuroradiologist #3	**0.719 [0.494–0.944]**	**0.830 [0.601–1.000]**	**0.667 [0.309–1.000]**
Senior diagnostic neuroradiologist #1	**0.644 [0.388–0.899]**	**0.791 [0.566–1.000]**	0.348 [−0.352–1.000]
Senior diagnostic neuroradiologist #2	0.595 [0.354–0.835]	**0.930 [0.794–1.000]**	0.340 [−0.089–0.768]
Senior diagnostic neuroradiologist #3	**0.771 [0.561–0.980]**	**0.927 [0.786–1.000]**	0.516 [−0.003–1.000]

*Bolded values indicate kappa values > 0.6, suggesting substantial to excellent agreement*.

Overall global judgment for the presence of blood and/or contrast across all specialties was fair, *k* = 0.291 (95% CI = 0.273–0.309) (Figure 1). There were 0 scans where the raters unanimously agreed on the presence of blood, contrast, neither, or both.

## Discussion

Our study shows that the overall inter-rater agreement about the presence of hemorrhage and/ or contrast was limited across all specialties. While DNR physicians seemed to have the highest degree of inter-rater agreement on the presence of hemorrhage, a *k* value of 0.495 still only represents a “moderate” level of agreement, and is usually considered “weak” in the context of health care research (7). However, intra-rater agreement for the presence of blood was consistently high across all specialties and levels of training, including junior physicians with <2 years of experience. This is in contrast with agreement on CS, where inter-rater agreement was only fair, and 100% of the physicians disagreed with themselves on their second reading at least once. Even though INR physicians were the most consistent in their judgment of contrast, their agreement was only deemed “moderate.” The lack of consensus on HT has been previously reported, and affects even simple dichotomized classifications such as hemorrhagic infarction vs. parenchymal hematoma (5), which are often used as outcome measures in clinical trials settings. While there are newly proposed rigorous classification systems for grading HT after ischemic stroke/reperfusion therapy, their reliability has not been assessed and they fail to address the issue of distinguishing CS from HT (8). The overall unreliable interdisciplinary interpretation of scans therefore may be attributed to unclear diagnostic criteria for CS and lack of additional imaging techniques to differentiate concurrent presence of both (9).

It is well-established that the phenomenon of CS can be seen after EVT, and is thought to relate to disruption of blood–brain barrier integrity in established ischemic infarct (10). Factors such as prolonged procedure time and multiple passes in the same vessel have been associated with higher risk for CS (11). The incidence of cerebral hyper-dense lesions after revascularization is high, and has been reported to be between 23 and 84%, depending on the definitions used and timing of follow-up imaging (10, 12). CS itself has been postulated to be associated with increased risk for HT and symptomatic ICH, although this is likely confounded by similar risk factors, such as large infarct size (13). Unfortunately, inaccurate detection of hemorrhage can lead to delayed initiation of anti-thrombotics, erroneous prognostication, and unnecessary investigations (9). It may be necessary to perform advanced imaging such as dual-energy CT (DECT) or gradient-recalled echo (GRE) sequence MRI for definitive diagnosis of hemorrhage vs. contrast (9, 14). However, MRI may be inaccessible to many centers in a timely fashion, and can still lead to false positive hemorrhage detection or false negative contrast extravasation if performed too soon after administration of contrast (14). DECT utilizes two distinctive voltage acquisitions to discriminate between materials with various attenuation properties, such as iodine vs. calcium or hemorrhage, but its availability is currently limited across centers. Future studies may look at the combination of concurrent SWI with CT and compare them to plain CT images for reference.

Our study has important limitations. Imaging assessments were done in controlled settings with no time constraints and therefore results may differ from real-time clinical assessments. Accuracy analysis was not performed because of the extensive disagreements revealed between each rater, thereby defeating the relevance of such. While diagnostic accuracy was not the goal of the study, one potential way to address the lack of a “gold standard” would be the use of advanced imaging (i.e., MRI susceptibility based images or dual-energy CT scans). We recognize that the pragmatic approach to resolving disagreements and addressing uncertainty in imaging interpretation is effective communication between specialties. Lastly, this case series of patients was artificially constructed to minimize paradoxes of *k* statistics, and the exact results might not be reproducible in a different case series of patients.

## Conclusion

There is a lack of agreement between physicians on the interpretation of post-EVT conventional CT scans for the presence/absence of both hemorrhage and contrast. Standardized definitions and clear diagnostic criteria for the two entities are warranted. Advanced imaging modalities such as DECT may be helpful in differentiating the two, if clinically indicated.

## Data Availability Statement

Access to the data that support the findings of this study are available from the corresponding author upon reasonable request.

## Ethics Statement

The studies involving human participants were reviewed and approved by Fondation Rothschild Ethics Committee. Written informed consent for participation was not required for this study in accordance with the national legislation and the institutional requirements.

## Author Contributions

RL was responsible for performing the statistical analysis and writing the manuscript. RF was responsible for study design and data collection. All authors contributed to the writing and editing of the manuscript for intellectual content.

## Conflict of Interest

The authors declare that the research was conducted in the absence of any commercial or financial relationships that could be construed as a potential conflict of interest.
